# Principal mechanisms of extracellular matrix‐mediated cell–cell communication in physiological and tumor microenvironments

**DOI:** 10.1111/febs.70207

**Published:** 2025-08-01

**Authors:** Zoi Piperigkou, Sylvia Mangani, Nikolaos E. Koletsis, Christos Koutsakis, Nicholas S. Mastronikolis, Marco Franchi, Nikos K. Karamanos

**Affiliations:** ^1^ Biochemistry, Biochemical Analysis and Matrix Pathobiology Res. Group, Laboratory of Biochemistry, Department of Chemistry University of Patras Greece; ^2^ Department of Otorhinolaryngology‐Head and Neck Surgery School of Medicine, University of Patras Greece; ^3^ Department for Life Quality Studies, University of Bologna Rimini Italy

**Keywords:** biomechanical sensing, cancer targeting, cell–cell communication, extracellular matrix, extracellular vesicles

## Abstract

Cell–cell communication is essential for the regulated exchange of information between cells, coordinating critical cellular processes under physiological and pathological conditions. The extracellular matrix (ECM) is a complex three‐dimensional (3D) intercellular macromolecular network that provides structural support to tissues, while actively modulating cellular functions and responses. ECM‐mediated intercellular communication is a key player in both homeostasis and disease development. Particularly in cancer, ECM reorganization drives tumor development and progression, shaping dynamic interactions within the tumor microenvironment (TME). In this review, we present and discuss two principal mechanisms of matrix‐mediated cell–cell communication in both physiological and cancerous contexts. First, we explore the impact of ECM biomechanical properties in mechanical sensing and communication, which govern key aspects of cell signaling, adhesion, and migration across normal and malignant tissues. Second, we discuss the role of the ECM in facilitating cell–cell communication through the controlled release and navigation of extracellular vesicles (EVs). EVs carry, among other constituents, proteins, enzymes, microRNAs (miRNAs), and signaling molecules that relay information to nearby or distant cells, modulating the initiation of metastasis and pre‐metastatic niche formation. Conclusively, in this review, we highlight the critical role of targeting ECM dynamics in cell–cell communication under physiological processes and during cancer progression. Targeted therapies that modulate ECM components and interactions with cells hold promise for future treatment approaches.

## Graphical abstract

Intracellular communication controls the processing of generated signals so that every cell type can successfully respond. The extracellular matrix (ECM) is a fundamental regulator of cell communication and signaling through the strict orchestration and functions of its bioactive components. As structural bioscaffolds, ECMs support the cell microenvironment and dictate cell functions, drive cell mechanosensing through force transmission, and mediate cell–cell intercellular communication via extracellular vesicles (EVs) release.

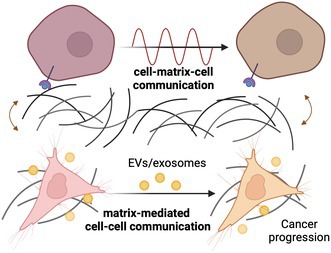

**Abbreviations**
3D: three‐dimensional
CAFs: cancer‐associated fibroblasts
CMACs: cell‐matrix adhesion complexes
E/M: epithelial/mesenchymal
ECM: extracellular matrix
EMT: epithelial‐to‐mesenchymal transition
ERs: estrogen receptors
EVs: extracellular vesicles
FAK: focal adhesion kinase
FGF: fibroblast growth factor
GAGs: glycosaminoglycans
GFRs: growth factor receptors
GFs: growth factors
GPCRs: G‐protein coupled receptors
HA: hyaluronan
HPSE: heparanase
miRNA: microRNA
MMPs: matrix metalloproteinases
MSC: mesenchymal stem cell
MVBs: multivesicular bodies
PGs: proteoglycans
PMN: pre‐metastatic niche
RTKs: receptor tyrosine kinases
TME: tumor microenvironment
TNBC: triple‐negative breast cancer
TNTs: tunneling nanotubes
VEGF: vascular endothelial growth factor

## Introduction

**Fig. 1:**
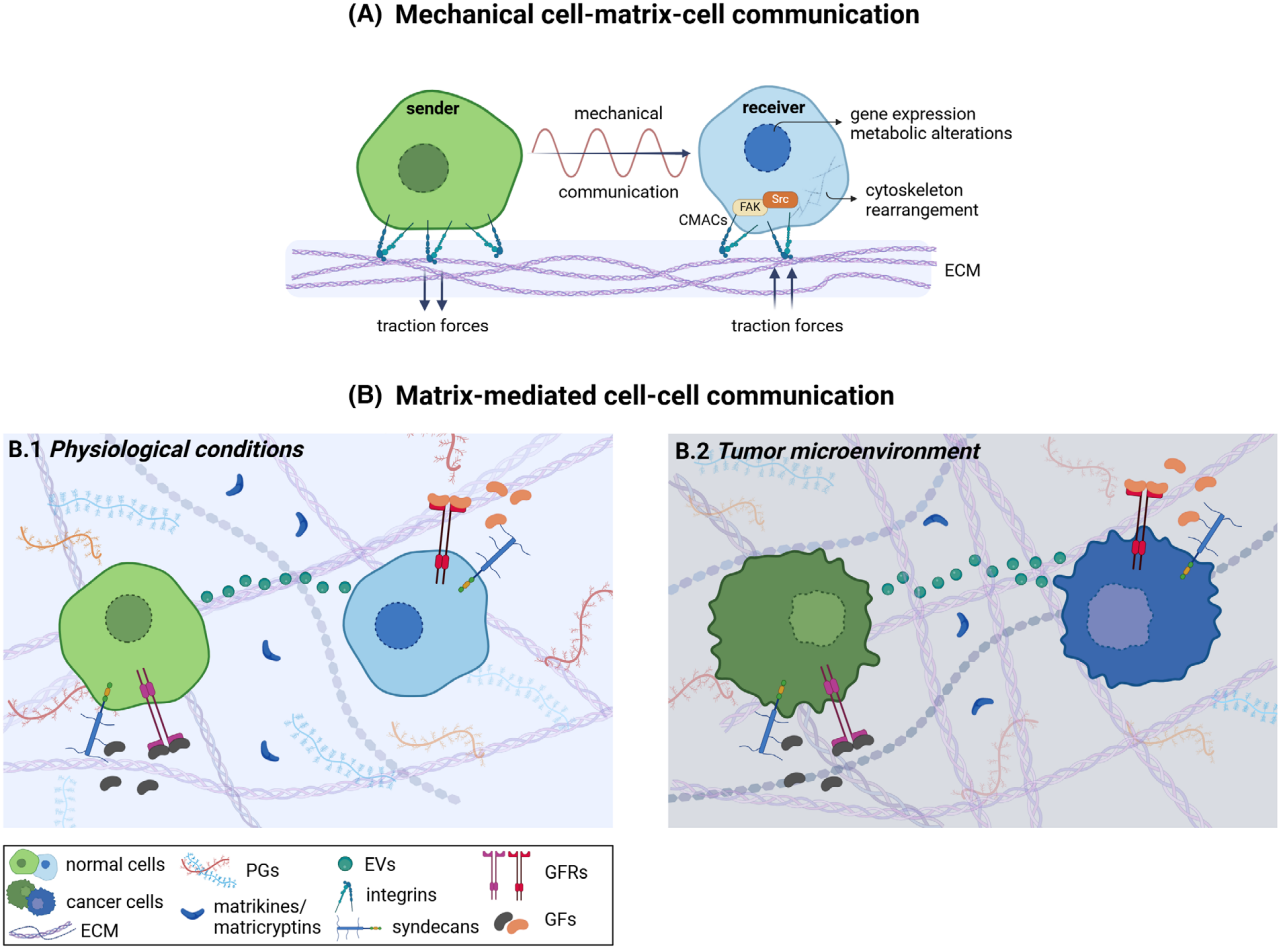
Mechanisms of matrix‐mediated intercellular communication. Schematic representation that demonstrates the types of  intercellular communication, focusing (A) on cell‐matrix‐cell communication through ECM‐mediated biomechanical sensing, and (B) matrix‐mediated cell–cell communication. (A) Cell‐matrix‐cell communication occurs through traction forces transmitted from a sender cell to a receiver cell. These forces are sensed by CMACs, and trigger downstream signaling pathways that regulate cellular adaptation, cytoskeletal rearrangement, and metabolic alterations in the receiver cell. Mechanical traction forces generated through ECM dynamics drive key physiological processes, including embryogenesis, organ development, wound healing, vascular assembly, synchronized cardiomyocyte beating, and also contribute to cancer progression. (B) Matrix‐mediated intercellular communication regulates downstream signaling pathways, mediating cellular phenotype and properties, under physiological conditions and cancer progression. (B.1) The ECM network drives cell–cell communication by serving as a reservoir for signaling molecules (i.e., GFs, EVs, matrikines, and matricryptins), which modulate downstream signaling cascades involved in various physiological processes, including, among others, wound healing and tissue regeneration, immune modulation, and homeostasis. (B.2) ECM assembly influences cell–cell communication, as matrix stiffening derived from aberrant accumulation of matrix components can lead to a pathological microenvironment that promotes cancer development and progression. Particularly, stiff ECM facilitates increased release of EVs and altered secretion of matrix mediators, thereby mediating cell–cell communication within the TME. CMACs, cell‐matrix adhesion complexes; ECM, extracellular matrix; EVs, extracellular vesicles; FAK, focal adhesion kinase; GFs, growth factors; GFRs, GF receptors; PGs, proteoglycans; Src, nonreceptor protein tyrosine kinase of Rous sarcoma virus c‐Src oncogene; TME, tumor microenvironment. Created in BioRender.

Cell communication is a fundamental biological process, allowing cells to respond to extracellular stimuli and share information to ensure proper function, growth, and immune responses. Intercellular communication is also a fundamental mechanism for the controlled flow of information between cells, coordinating critical pathophysiological cellular processes [1]. Cells have developed sophisticated and highly coordinated ways to communicate with each other and rapidly adapt to microenvironmental changes in both physiological and pathological conditions. Cells communicate through major mechanisms, such as direct contact via gap junctions, chemical signals (i.e., autocrine, paracrine and endocrine signaling) and electrical impulses (i.e., muscle contractions). Extracellular matrix (ECM) functions as three‐dimensional (3D) bioscaffold and signaling hub [2] serving intercellular communication in two main ways. First and foremost, ECM biomechanical support creates dynamic oscillation phenomena that drive cell‐matrix‐cell communication through cell surface receptors, such as integrins. Importantly, ECM conformation is not static, but it constantly undergoes controlled remodeling that is stimulated by cellular responses, environmental cues, and biomechanical stimuli, often resulting in rhythmic changes in its biochemical composition and biomechanical properties (Fig. 1A). The dynamic interplay among cancer cells and the peri‐tumoral ECM is also driven by cell mechanosensing and mechanotransduction mechanisms, which trigger signaling cascades that regulate cancer growth and development [3, 4]. On the contrary, ECM constituents, including macromolecules and released vesicles, mediate cellular responses via matrix‐mediated cell–cell communication (Fig. 1). These insights are pivotal in guiding the development of ECM‐targeted therapies aiming at modulating aberrant signaling in disease contexts. Understanding these communication pathways enables the precise targeting of the ECM to restore tissue homeostasis or inhibit cancer progression. Disrupting pathological ECM remodeling and deciphering ECM‐mediated interactions is central to translating medicine into effective clinical interventions.

The integral and active matrix components are fundamental parts in all tissues and organs, affecting cellular behavior and interactions (i.e., communication and signaling) [5]. ECMs constitute 3D dynamic complex intercellular macromolecular networks, consisting of collagens and elastin, proteoglycans (PGs), glycosaminoglycans (GAGs) and glycoproteins, such as fibronectin and laminins. Acting as structural 3D bioscaffolds, ECMs provide physical structure and mechanical support and also dictate functions of resident cells within tissues (Fig. 1A) [6]. Importantly, the composition of each ECM determines cell morphology and, consequently, cell communication. Under physiological conditions, the synthesized ECM constantly undergoes remodeling processes that maintain tissue homeostasis. ECM serves as a reservoir of molecules and vesicles that promote either direct cell–cell interactions or cell‐matrix interactions. These molecules also constitute signals that induce intercellular transduction cascades, regulating both normal cellular processes (Fig. 1B.1) and also the initiation and progression of various diseases [7, 8, 9].

In several pathological conditions, including cancer, however, ECM synthesis and turnover are dysregulated, leading to changes in its integrity [10]. During cancer development and progression, mechanical and biochemical cues from the ECM are detected through cell receptors and play important roles in promoting ECM‐cell intercellular signaling (Fig. 1B.2) [3]. ECM–cancer cell interactions shape the pre‐metastatic niche (PMN) by influencing cell adhesion, migration, and the release of signaling molecules that initiate metastatic outgrowth. Protease‐induced limited cleavage of matrix components can trigger the secretion of bioactive fragments exerting different actions from those of the full‐length molecules [11]. Matricryptins and matrikines are the bioactive fragments released from PGs and GAGs and are reported to exert major roles in modulating signaling cascades mediated by interactions with cell surface receptors [12, 13]. Moreover, syndecans, a family of cell surface heparan sulfate PGs, apart from the proteolytic shedding, could be processed by the glycolytic enzyme heparanase (HPSE), thereby modulating growth factor availability and downstream signaling cascades. These fragments may regulate several physiological and pathological processes, including wound healing, angiogenesis, fibrosis, and cancer [13, 14].

**Fig. 2:**
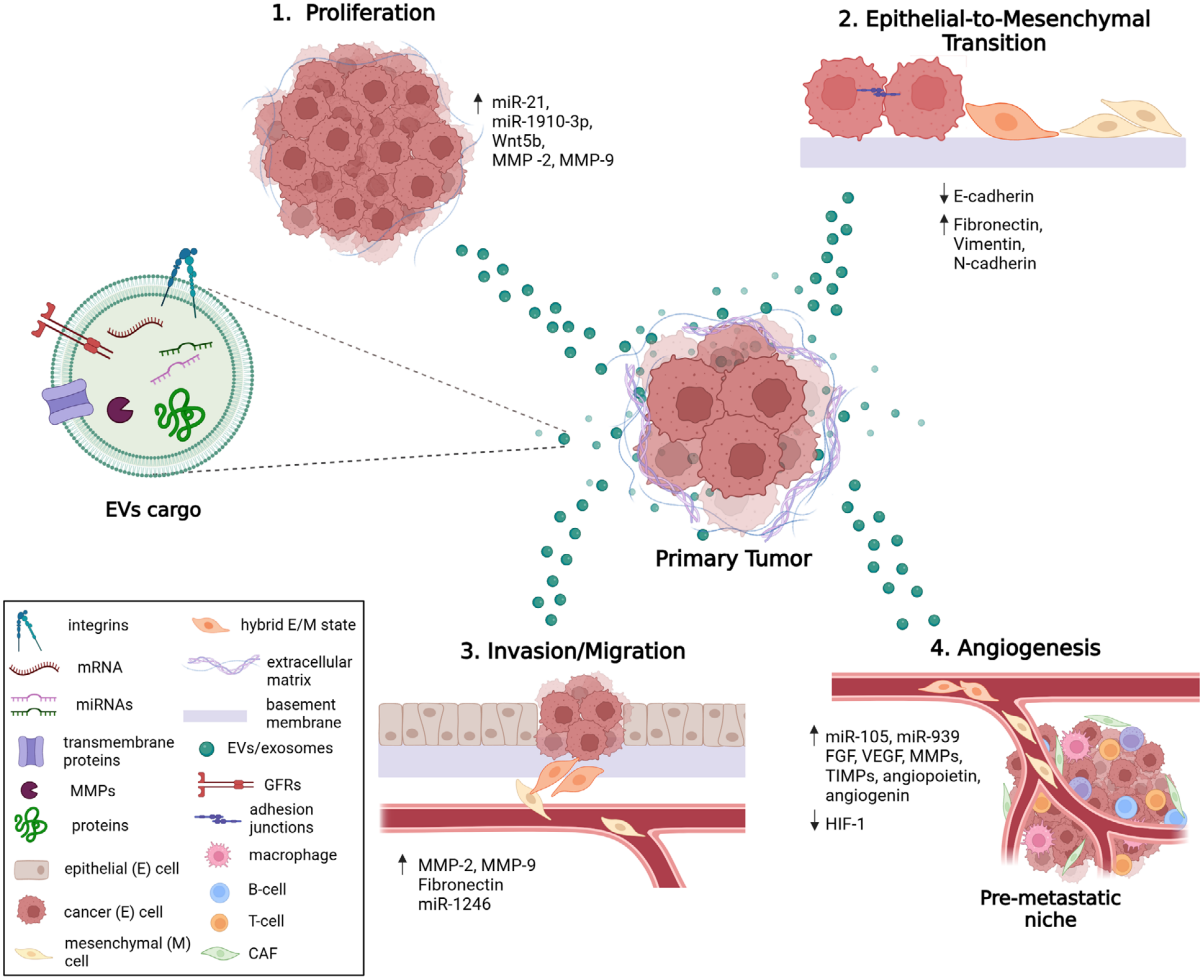
Multifaceted roles of cancer cell‐derived extracellular vesicles (EVs) in driving cancer progression. Cell–cell and cell‐matrix communications via EVs cargos affect cell functional properties (1, 3, 4) and morphology (2), facilitating cancer development and propagation. EVs enriched in bioactive molecules, such as mRNA, miRNAs, transmembrane proteins, proteolytic enzymes, membrane receptors (i.e., GFRs and GPCRs), and co‐receptors (i.e., integrins), are released from cancer cells into the surrounding ECM. Upon uptake by recipient cells, EVs cargos modulate signaling pathways involved in crucial cellular processes, including proliferation (1), EMT (2), invasion/migration (3), and angiogenesis (4). To this end, the secreted vesicles influence cancer cells and other cell types within the TME, thereby facilitating PMN formation in secondary tissues and the initiation of metastasis. Additionally, cancer cell‐derived EVs are key players in ECM remodeling by promoting ECM degradation and facilitating the establishment of a supportive microenvironment for cancer growth and dissemination. CAF, cancer‐associated fibroblast; ECM, extracellular matrix; EVs, extracellular vesicles; EMT, epithelial‐to‐mesenchymal transition; FGF, fibroblast growth factor; GFRs, growth factor receptors; GPCRs, G‐protein coupled receptors; HIF‐1, hypoxia‐inducible factor 1; mRNA, messenger RNA; miRNA, microRNA; MMP, matrix metalloproteinase; PMN, pre‐metastatic niche; TIMP, tissue inhibitor of metalloproteinases; TME, tumor microenvironment; VEGF, vascular endothelial growth factor. Created in BioRender.

The dynamic oscillatory nature of ECM plays a crucial role in intercellular communication by modulating extracellular vesicle (EV) production and release (Fig. 1B). EVs are membrane‐bound nanoparticles, released by cells into the ECM. They are classified into three categories: the exosomes (30–250 nm in diameter) and the microvesicles (100–1000 nm), which control intercellular communication, and the apoptotic bodies (1–5 μm) [15]. Their cargo, including proteins, lipids, nucleic acids (i.e., mRNA, miRNA, and DNA), and metabolites, influence recipient cell behavior. EVs production is tightly regulated by ECM dynamics, such as stiffness, composition, and enzymatic remodeling. EVs have a distinct position in health and disease as they exhibit their effects in both physiological and pathological conditions [16]. In physiological conditions, they contribute to tissue repair, immune modulation, and homeostasis. In cancer, the ECM‐driven EV release enhances tumor progression by transferring oncogenic signals, modulating immune responses, and facilitating PMN formation. Notably, proteolytic enzymes like matrix metalloproteinases (MMPs) and HPSE remodel the ECM, altering EV composition and cargo loading [17, 18]. Additionally, ECM interactions guide EV uptake by recipient cells, influencing processes, such as proliferation, invasion, and chemoresistance [19]. Tumor‐derived EVs transfer oncogenic proteins, enzymes, and miRNAs that promote proliferation, epithelial‐to‐mesenchymal transition (EMT), angiogenesis, migration/invasion (Fig. 2). EVs carrying integrins (i.e., α6β4 and ανβ5) prime specific organs for metastasis via interacting with ECM components. Importantly, miR‐1 and miR‐29a have been identified as key oncogenic miRNAs in tumor‐derived EVs, promoting tumor progression and immune suppression [20]. Likewise, EVs may suppress tumor growth by delivering tumor‐suppressive molecules or activating antitumor immune responses, as in the case of immune cell‐derived EVs carrying tumor antigens and stimulating antitumor T‐cell responses. These ECM‐regulated EV mechanisms play a pivotal role in shaping the tumor microenvironment and driving metastasis (Fig. 1B).

In this article, therefore, we present and critically discuss the role of ECM tension/traction forces in establishing processes that facilitate mechanical intercellular cell‐matrix‐cell communication. The matrix‐mediated cell–cell communication in cancer progression is also discussed. The essential roles of matrix dynamics in regulating EV/exosome secretion, transfer, and cargo composition in EV‐mediated cell–cell communication within the tumor microenvironment (TME) are thoroughly presented and discussed.

## ECM biomechanical properties drive cell–cell communication

ECM supports cell communication, adaptation, and response to extracellular stimuli by facilitating cell‐matrix‐cell interactions and mediating matrix‐driven cell–cell communication in physiological and pathological conditions (Fig. 1). The functional roles of major ECM macromolecules under physiological and pathological conditions are summarized in Table 1 (i.e., wound healing, tissue development) and Table 2 (i.e., cancer progression), respectively.

**Table 1:** Functional roles of major matrix components and extracellular matrix‐related effectors in intercellular communication under physiological processes. BMP, bone morphogenic protein; CXCL, chemokine (C‐X‐C motif) ligand. EGF, epidermal growth factor; FGF, fibroblast growth factor; HA, hyaluronan; HSPGs, heparan sulfate proteoglycans; IGF, insulin‐like growth factor; ICAM, intercellular adhesion molecule; IL, interleukin; MMPs, matrix metalloproteinases; OPN, osteopontin; PDGF, platelet‐derived growth factor; PF4, platelet factor; RSPO1, R‐spondin 1; TGF, transforming growth factor; THBS, thrombospondin; TNF, tumor necrosis factor; VCAM, vascular cell adhesion molecule; VEGF, vascular endothelial growth factor.

Physiological process	ECM component	Functional role	References
Wound healing	Collagen type I, III; fibronectin; integrin αIIbβ3	Homeostasis; platelet aggregation	[69, 70, 71]
	Collagen type I, IV, XVII; laminin α5; laminin 511; laminin 521; perlecan; CCN1; CCN3; CCN5	Keratinocyte activation, proliferation, and migration	[72, 73]
	Collagen type VII	Fibroblast and keratinocyte migration; granulation tissue formation	[72]
	Tenascin‐C	Lymphocytes and fibroblasts activation and migration	[72]
	Collagen type I; fibronectin; vitronectin; THBS	Provisional matrix; fibroblast and leukocyte migration	[74]
	HA	Fibroblasts migration to the wound site	[75]
	Integrins β1, β2	Leukocytes, keratinocytes, and fibroblasts recruitment to the wound site; myofibroblast differentiation and blood vessel sprouting	[74, 76]
	MMPs	Fibroblasts‐released enzymes; immune cell attraction and infiltration; Macrophage‐released enzymes; laminin cleavage, binding of the fragment to EGFR on fibroblasts; stimulation of keratinocytes migration and proliferation	[77, 78]
	Nidogen 1	Cell proliferation and differentiation during re‐epithelialization	[72]
	Fibronectin; matrikines	Chemoattractants for monocytes	[32]
	OPN	Chemoattractant for fibroblasts	[72]
	Fibronectin; HA	Early granulation tissue formation	[32]
	THBS1; THBS4; periostin; RSPO1	Fibroblasts, keratinocytes proliferation and migration	[73]
	VCAM; ICAM1; P‐selectin E‐selectin	Neutrophil adhesion onto endothelial cells; neutrophil extravasation from the bloodstream to the wound site	[79]
	ICAM1	Activated fibroblasts stimulate dendritic cells	[77]
	Laminin 332	Re‐epithelialization; ECM remodeling by keratinocytes	[80]
Tissue formation and development	Collagen type XII	Tendon development; intercellular communication by building matrix bridges in between adjacent tenocytes	[81]
	Perlecan	Neurons, muscles, glia, fat body, and hemocytes communication; axonal and synaptic stability during development	[82]
	E‐cadherin	Gonad development (germ and somatic cells interactions); osteoclastogenesis	[83, 84]
	N‐cadherin; cadherin‐11	Bone formation; osteoblast differentiation	[85]
	Fibronectin	(integrin‐mediated) cellular migration and differentiation during lung development	[76]
	HSPGs	Morphogens/growth factors presentation to cell surface receptors; development of central neural and musculoskeletal system	[86]
**ECM‐related effectors**			
Wound healing	PF4	Platelet‐released cytokine; chemotaxis of fibroblasts, neutrophils, monocytes; differentiation of monocytes into macrophages	[87]
	PDGF; TGF‐β	Platelet‐released factor; chemotaxis and proliferation of immune cells, fibroblasts, smooth muscle cells	[32, 79, 88]
	TGF‐β1	Platelet‐released factor; stimulates keratinocyte proliferation	[89]
	IL‐1α, IL‐1β, IL‐6, IL‐8; CXCL8; TNF‐α	Platelet‐released factors; migration of neutrophils, macrophages, mast cells	[90]
	VEGF; TGF‐α; bFGF	Platelet‐released factors; activation of endothelial cells (angiogenesis)	[79]
	VEGF; PDGF; bFGF; TGF‐β	Endothelial cells‐released factors; fibroblasts proliferation and migration	[91]
	EGF; TGF‐α	Μacrophages, platelets, keratinocytes‐derived factors; re‐epithelialization	[32]
Tissue formation and development	FGF4	Cell–cell communication; differentiation of epiblast‐like and primitive endoderm‐like cells	[92]
	FGF; EGF; VEGF	Embryogenesis; communication between nourishing ectodermal cells and endometrial cells during implantation	[93]
	VEGF; IGF	Endometrial angiogenesis	[93]
	BMP‐2, BMP‐4, BMP‐7; IGF	Endothelial cells‐released factors; osteoblasts, osteoclasts, bone marrow‐derived mesenchymal stem cells activity in osteogenesis	[94]
	VEGF‐A; PDGF‐BB	Osteoblasts, osteoclasts and bone marrow‐derived mesenchymal stem cell‐released factors; regulation of proliferation and migration of endothelial cells; angiogenesis	[94]

**Table 2:** Functional roles of major matrix components and extracellular matrix‐related effectors in intercellular communication in cancer progression. CCL, chemokine (C‐C motif) ligand; CXCL, chemokine (C‐X‐C motif) ligand; FGF, fibroblast growth factor; GM‐CSF, granulocyte‐macrophage colony‐stimulating factor; GPC, glypican; HIF, hypoxia‐inducible factor; ICAM, intercellular adhesion molecule; IL, interleukin; LIF, leukemia inhibitory factor; LOX, lysyl oxidase; OPN, osteopontin; PDGF, platelet‐derived growth factor; SDC, syndecan; TGF, transforming growth factor; uPA, urokinase plasminogen activator.

Pathological process	ECM component	Functional role	References
Cancer progression	Integrins	Cell–ECM interactions via focal adhesions and hemidesmosomes; ECM remodeling, cell migration, cancer stemness	[26, 39, 95]
	Fibronectin	Fibronectin fibers alignment by CAFs; cancer cell migration	[95]
	SDC‐1	Fibroblasts‐derived SDC‐1 organizes ECM fibers in parallel arrays; cancer cells migration and invasion	[96, 97]
	SDC‐2 (ectodomain)	Cancer cell‐released SDC‐2 (shed); macrophages attraction to the tumor and conversion into TAMs; endothelial cells activation to induce angiogenesis	[98]
	GPC‐3	Cancer cell PG; recruitment of M2‐polarized macrophages	[99]
	Versican	Cancer cells‐secreted PG; regulates macrophage function CAF‐released PG; cancer cell migration/invasion	[100, 101]
	Lumican	CAF‐released PG; tumorigenesis and metastasis	[43]
	ICAM1	CAFs‐released molecule; immune cell activation and polarization; cancer cell quiescence and drug resistance	[102]
	MMP‐1, MMP‐3	Fibroblast‐released enzymes; E‐cadherin cleavage, leading to cancer cells EMT and invasion	[102]
	MMP‐2, MMP‐9, MMP‐13, MMP‐14	Cancer cell‐released enzymes; inhibition of T‐cell proliferation and antigen presentation	[103, 104]
	uPA	CAF‐released enzyme; cancer cell migration/invasion	[43]
	Collagen, LOX	Cancer cell‐derived enzyme; ECM stiffness; cancer cell pseudopodia formation, enhanced proliferation and spreading; cancer cell metabolic reprogramming	[105, 106]
	OPN	Cancer cell‐released protein; stromal cell recruitment	[107]
	Tenascin‐C	CAF‐released glycoprotein; cancer cell proliferation, migration	[43]
**ECM‐related effectors**			
Cancer progression	HIF	Cancer cells‐released factors; macrophage and fibroblast recruitment to hypoxic regions of the primary tumor; ECM remodeling, angiogenesis	[95]
	HGF, CTGF, EGF, FGF, IGF, TGF‐β, CXCL11, CXCL12, IL‐6, IL‐10, IL‐1β	CAF‐released factors; cancer cell survival, proliferation, invasion	[101, 102, 108, 109]
	VEGFA, TGF, PDGF, IL‐6, CXCL12	CAF‐released factors; angiogenesis, recruitment of bone marrow‐derived endothelial cells	[102, 108]
	IL‐6, IL‐17, VEGF, FGF, TNF‐α	Bone marrow‐mesenchymal stem cells‐released factors; cancer cell survival, proliferation and drug resistance	[110]
	TGF‐β	Cancer cell‐released factor; fibroblasts stimulation, collagen and fibronectin production, chemokines (related to tumor promotion) secretion	[111]
	GM‐CSF, LIF	Cancer cell‐ and CAF‐released factors; macrophage recruitment and polarization	[112]
	CCL2, CCL5	CAF‐released cytokines; macrophage attraction	[108]
	TGF‐β, IL‐6	CAF‐released cytokine; cancer cell EMT	[43, 108]

### Cell communication in physiological processes

Within the tissues, cells sense, modify, and adapt to their microenvironment across a wide range of physiological and pathological biological processes by generating forces, allowing for mechanical cell–cell interactions [21, 22, 23, 24]. Particularly, long‐range ECM‐mediated cell–cell force transmission facilitates the communication between adjacent or distant cells, determining their morphology, motility, and functional properties [21, 25]. Notably, cells exert dynamic traction forces on the surrounding matrix, acting either as transmitters or receivers of mechanical signals [21, 24, 26] (Fig. 1A). The mechanical stimuli are translated through cell‐matrix adhesion complexes (CMACs) into intracellular signaling pathways, ultimately driving downstream cytoskeleton rearrangements, metabolism alterations, or regulating target gene expression [27]. CMACs are highly dynamic complexes formed by integrin clustering, along with the recruitment (and downstream phosphorylation) of cytoplasmic proteins that bind to the cytoskeleton [28]. Various cellular functional properties, such as differentiation, proliferation, and migration, rely on matrix mechanical properties [22] (Fig. 1A). Mechanical cell‐matrix‐cell communication drives various physiological processes, including collective cellular motility during morphogenesis, embryogenesis, organ development, and multicellular organization, wound healing, and vascular assembly [29, 30] (Fig. 1B.1; Table 1). ECM molecules, along with bioactive ECM‐related effectors, orchestrate cell–cell signaling, essential for wound healing, embryogenesis, and tissue/organ development. Cells realign in response to external strain, while mechanical interactions mediated through matrix strain propagation can synchronize their alignment [24].

During tissue development, cells sense mechanical signals through integrins (Table 1). Mechanical communication between endothelial cells, driven by stress gradients and ECM strain stiffness, further influences vascular network formation. Particularly, stiffness gradients and local fiber alignment guide endothelial cells toward each other before forming stable vascular endothelial cadherin‐enriched junctions [24, 29, 31]. During vascularization, mechanical intercellular communication is strongly mediated through focal adhesion kinase (FAK) and mechanosensitive ion channels, inducing downstream calcium signaling [29]. It is well known that upon tissue injury, the ECM undergoes extensive remodeling, facilitated by the action of MMPs and other proteolytic enzymes which create paths for cell migration and thus enable communication between fibroblasts, endothelial cells, and other cell types (Table 1). Furthermore, ECM remodeling releases sequestered growth factors (GFs), cytokines, and other signaling molecules, enabling them to interact with their respective receptors on target cells and modulate cellular responses [32] (Fig. 1B.1; Table 1). Additionally, during the inflammatory phase of wound healing, fibroblasts exert traction forces within their surrounding ECM to facilitate wound closure. This process is particularly regulated by mechanotransducers (including FAK and YAP/TAZ mechanosensors) and integrins that drive proto‐myofibroblast formation [25] (Table 1). These mechanical signals extend beyond chemotactic gradients, inducing FAK downstream pathways that hold key roles in myofibroblast differentiation and macrophage migration [25, 33]. Interestingly, Pakshir *et al*. demonstrated that fibroblasts can create deformation fields in the fibrillar collagen matrix, generating long‐range mechanical/physical cues that drive macrophage migration. Macrophages sense and respond to these mechanical signals through α2β1 integrin binding and stretch‐activated channels [34]. Furthermore, Sunyer *et al*. [35, 36] revealed that collective cell migration, occurring during development, wound healing, or even cancer progression, is guided by long‐range intercellular forces transmitted through gradient‐stiffness substrates.

Mechanotransduction mechanisms guided by matrix modulators, including integrins, receptor tyrosine kinases (RTKs), G‐protein coupled receptors (GPCRs), stretch‐activated ion channels, and the subsequent signaling molecules (i.e., FAK and Src) enable cells of different origins to interact with ECM. To this end, cell membranes actively change conformation by pulling or pushing, often via actin polymerization and microtubule formation. For instance, high molecular weight hyaluronan (HA), the most distributed nonsulfated GAG in the majority of ECMs, is related to the proper lubrication and water homeostasis in several tissues [37]. Understanding the dynamic biosensing and adaptation functions of the matrix contributing vital physiological functions has crucial implications in biomedical research, and it has been further revised in critical reviews [3, 14].

### Cell communication in cancer progression

Mechanical cell–cell communication holds a crucial role under pathological conditions, particularly cancer progression [30] (Fig. 1B.2). Within the TME, mechanical forces are closely associated with tumor growth, invasion, angiogenesis, and drug resistance [38]. Particularly, excessive cancer cell proliferation, combined with elevated interstitial fluid pressure resulting from tumor‐driven angiogenesis, generates circumferential mechanical tension forces at the invasive edge of the tumor mass [39]. Cancer cells communicate with neighboring cells by transmitting mechanical forces through the ECM via CMACs [40]. Mainly integrins, but also surface PGs, cadherins, Ca^2+^ channels, and stretch‐activated cation channels can detect matrix biophysical properties, such as ECM stiffness [41] (Table 2). Notably, cellular forces exerted onto the surrounding ECM lead to matrix remodeling, thereby inducing fiber alignment. Cells in contact with the surrounding matrix sense these signals through their CMACs, resulting in activation of downstream signaling pathways and consequent phenotypic changes [26].

On the contrary, cancer‐associated fibroblasts (CAFs) hold a crucial role in cancer progression by remodeling the basement membrane and releasing matrix‐degrading enzymes that create microtracks in the ECM, enabling cancer cells to migrate from the primary tumor and invade surrounding tissues [26, 41, 42, 43]. Particularly, CAFs form mechanically active adhesions with cancer cells, exerting pulling forces during contraction or migration to further drive cancer cell invasion (Fig. 2). This process heavily relies on ECM proteins and integrins to transmit tension, highlighting the important role of cell‐matrix‐cell communication in cancer progression and metastasis [44] (Table 2). Furthermore, ECM stiffness alterations (accumulation/remodeling), driven by enzymes released from cancer cells and fibroblasts, promote sprouting angiogenesis, enabling endothelial cells to migrate and form vascular networks/capillaries [26] (Fig. 2; Table 2).

## ECM regulates extracellular vesicle release and cell–cell communication during cancer progression

ECM plays a crucial role in regulating the release and content of EVs, as well as influencing their interaction with and navigation through the ECM. The internal content of EVs, the surface‐associated macromolecules, and the EV‐evoked signaling effects in target cells define the bioactivity of EVs. EVs can interact with target cells by binding to cell surface receptors or by being internalized. Additionally, they may participate in ECM remodeling by carrying proteolytic enzymes, such as MMPs, or by regulating the expression of ECM macromolecules in target cells (Fig. 2). Recent studies have demonstrated that the ECM mechanical properties, particularly its stiffness, can also regulate the secretion of EVs [45]. Cells cultured on stiffer ECM substrates secrete higher levels of EVs compared to those on softer substrates, a phenomenon mediated by mechanotransduction pathways that translate ECM stiffness into intracellular signaling cascades, ultimately affecting the biogenesis and release of EVs [46]. In addition to modulating EV secretion, the ECM can also affect the cargo of EVs (Fig. 2). As shown in Table 2, ECM macromolecules, such as collagens, laminins, and fibronectin, can be selectively packaged into EVs and influence the behavior of recipient cells, contributing to processes, including tissue remodeling, cell migration, and angiogenesis. Furthermore, ECM remodeling can influence the uptake and biodistribution of EVs within tissues by creating physical barriers or facilitating the diffusion of EVs, thereby affecting their ability to reach and interact with target cells [47]. Finally, ECM components can serve as binding sites for EVs, modulating their localization and availability for uptake by recipient cells (Fig. 2).

Cancer initiation strongly depends on microenvironmental cues originated from various components of the niche, including the ECM, while communication between cancer and neighboring normal cells is fundamental for the progression of the disease [48, 49] (Fig. 2). Notably, ECM properties can influence intracellular signaling pathways as well as TME remodeling through secreted exosomes [50, 51]. Exosomes, an important subtype of EVs, are formed within endosomal compartments (i.e., multivesicular bodies, MVBs) and released via exocytosis, while their typical size is 30–150 nm. As functional matrix components, exosomes are involved in cell–cell communication, carrying bioactive components that mediate crosstalk between different cell types within the TME [52]. Exosomes regulate critical processes during cancer progression, including cell proliferation, angiogenesis, and PMN formation [53].

**Fig. 3:**
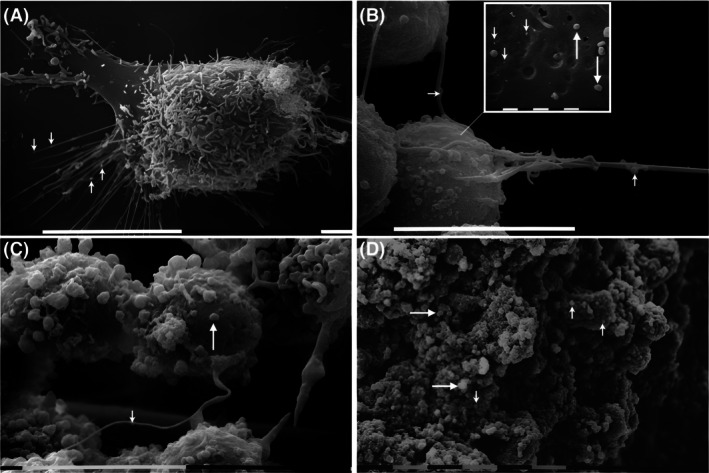
Scanning electron microscopy of 2D/3D cultures of breast cancer cells showing EVs release. (A) MDA‐MB‐231 breast cancer cells in 2D monolayers show many microvilli or short filopodia which release exosomes (arrows) in the microenvironment. White bar, 1 μm. (B) Three MDA‐MB‐231 breast cancer cells in 2D conditions are connected by TNTs on which exosomes are surfing (arrows). White bar, 1 μm. Inside the panel, a single MDA‐MB‐231 breast cancer cell at higher magnification displays exosomes less than 150 nm in diameter (small arrows) and microvesicles measured about 1000 nm (large arrows) on the cytoplasmic surface. White bar, 1 μm. The small pores showing different sizes at the cell surface correspond to the cytoplasmic sites of uptake and release of EVs. (C) MDA‐MB‐231‐derived 3D spheroids hold cells producing both exosomes and microvesicles. Cytoplasmic pores for the direct uptake of the exosomes are also visible (large arrow). A single exosome (small arrow) is surfing on the surface of a TNT connecting adjacent cells. (D) shERβ MDA‐MB‐231‐derived 3D spheroids produce a few exosomes (small arrows) and many microvesicles (large arrows), which are detectable on the cytoplasmic surfaces that display strict cell–cell contacts and no cytoplasmic protrusions or TNTs. The very compact spheroid induces the development of relatively wide intercellular channels (10 μm in diameter) which can ensure nutrient support to the inner cells of the spheroid. White bars, 10 μm. Scale bars are colored (white, black, or gray) for visibility only; gray indicates a white bar obscured by reduced brightness. Colors do not convey additional parameters beyond length. 2D, two‐dimensional; EVs, extracellular vesicles; TNTs, tunneling nanotubes.

Our group has established the key role of estrogen receptor beta (ERβ) in modulating aggressive phenotype, EMT, matrix composition, miRNA signatures, and tumorigenesis of triple‐negative breast cancer (TNBC) cells by silencing the expression of the functional ERβ1 variant [54, 55, 56]. Ιn Fig. 3, we demonstrate the alterations in morphological characteristics and EVs release between MDA‐MB‐231 TNBC breast cancer cells and ERβ‐suppressed cells (shERβ) in 2D and 3D cell cultures. As shown in Fig. 3A, SEM analysis of MDA‐MB‐231 cells in 2D monolayers demonstrates cellular protrusions (i.e., microvilli and filopodia), which release exosomes and microvesicles on the cytoplasmic sites of cell surface (Fig. 3B insert). Notably, the existence of filopodia and tunneling nanotubes (TNTs) that is observed in Fig. 3B has been previously associated with adhering exosomes and microvesicles surfing from one cell to another [57]. MDA‐MB‐231‐derived 3D spheroids, both microvesicles and exosomes are released in cell surface (Fig. 3C). The cytoplasmic pores for the direct uptake of EVs can also be observed (large arrows in Fig. 3C). On the other hand, in shERβ‐derived 3D spheroids (Fig. 3D), a lot of microvesicles and few exosomes can be observed. Moreover, cells are very compact but show intercellular channels probably avoiding the nutrient support in the spheroid core. These observations highlight the critical importance of ERs in modulating EVs release that in turn mediate cell–cell communication [7, 8].

Exosomal cargo may include ECM components modulating matrix assembly and subsequently impacting cell phenotype and communication [58, 59]. Abnormal deposition of ECM components may lead to the reorganization of the extracellular microenvironment, generating a cancer‐supporting matrix guiding the formation of PMN toward the initiation of metastasis [53, 60]. For example, exosomes from metastatic breast cancer cells activate MMP‐2, remodeling the ECM and inducing the release of specific GFs, resulting in invasion and metastasis [61] (Fig. 2). Interestingly, increased ECM stiffness has been associated with upregulation of exosome secretion, inducing intrinsic oncogenic signaling in recipient cells [50]. Syndecans have been shown to function as key players in membrane vesicle trafficking, guiding exosome biogenesis, composition, and release [62]. Fibronectin‐containing exosomes further regulate the functional properties of cancer cells, such as invasion and migration [63]. Additionally, exosomal integrins interact with ECM components, such as fibronectin and laminins, to promote local secretion of exosomes, facilitating adhesion and directed cell migration [16, 64] (Table 2).

**Fig. 4:**
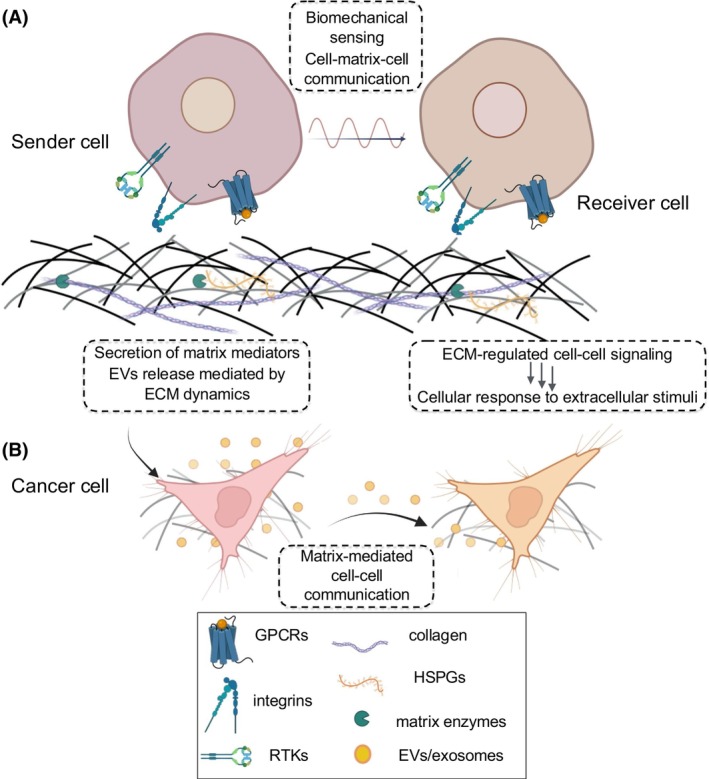
Emerging therapeutic strategies targeting matrix mechanosensing and intricate cell communication signaling. ECM is a genetically encoded and post‐translationally modified 3D macromolecular network that orchestrates cell‐matrix and matrix‐mediated cell–cell communication through mechanosensing signaling. (A) Biomechanical cues transmitted through the ECM and cell surface receptors (i.e., integrins, RTKs, GPCRs, and CMACs) influence cell behavior, contributing to tissue homeostasis or, under pathological conditions, promoting cancer progression, inflammation, and fibrosis. (B) ECM dynamics regulate the secretion of matrix mediators (i.e., growth factors, cytokines etc.) and the release of EVs/exosomes, which encapsulate key matrix components and serve as diagnostic biomarkers and potential therapeutic agents. Cancer cells exploit these ECM‐driven communication routes to support EMT, phenotypic switch, proliferation, migration/invasion, and angiogenesis in order to guide the formation of premetastatic niche and the initiation of metastasis. Therapeutic strategies include engineering EVs with ECM‐modifying cargo (i.e., siRNAs, miRNAs, protein modulators) to reprogram TME, and targeting specific intracellular pathways to influence EV secretion and function. 3D, three‐dimensional; CMACs, cell‐matrix adhesion complexes; ECM, extracellular matrix; EMT, epithelial‐to‐mesenchymal transition; EVs, extracellular vesicles; GPCRs, G‐protein‐coupled receptors; HSPGs, heparan sulfate proteoglycans; miRNA, microRNA; RTKs, receptor tyrosine kinases; siRNA, small interfering RNA; TME, tumor microenvironment. Created in BioRender.

Exosomes released from cancer cells are also enriched in ECM‐degrading enzymes that mediate matrix remodeling, thus promoting cancer cell invasion, dissemination to distant tissues, and PMN formation (Fig. 4) [4, 16]. For instance, exosomes from metastatic breast cancer cells have been shown to activate MMP‐2, thus remodeling the ECM and inducing the release of specific GFs, resulting in invasion and metastasis [61] (Fig. 2, Table 2). The following pro‐angiogenic proteins were identified in exosomes originating from glioblastoma cells: angiopoietin, angiogenin, fibroblast growth factor (FGF), vascular endothelial growth factor (VEGF), IL‐6 and IL‐8, and the tissue MMP inhibitors TIMP‐1 and TIMP‐2. Extensive studies in melanoma‐, myeloma‐, glioblastoma‐, and nasopharyngeal cancer‐derived exosomes have identified that angiogenesis is also boosted by exosomes enriched in MMP‐2, MMP‐9 and MMP‐13 [65]. Moreover, miRNAs carried by exosomes are involved in intercellular communication and promote angiogenesis, TME remodeling, and metastasis [16, 66]. Hence, exosomal miRNAs derived from cancer cells (i.e., miR‐21, miR‐23, miR‐30, miR‐105, miR‐130, miR135, miR‐155, miR‐210, miR‐424, and miR‐939) act as mediators between cancer cells and distant tissues, inducing paracrine or endocrine pathways to regulate cancer cell behavior [67, 68]. Exosomal miRNAs also induce MMP expression and promote EMT, thereby enhancing the metastatic potential of cancer cells [16].

## Conclusions and future perspectives

ECMs are unique, genetically encoded, and post‐translationally modified 3D dynamic macromolecular networks of interacting macromolecules that play critical roles in regulating tissue homeostasis. Extracellular stimuli sense ECM (Fig. 4A) and establish a mechanism of cell‐matrix and cell–cell mechanical communication, driving the progression of pathological conditions, including cancer, inflammation, and fibrotic diseases via endocrine and paracrine interactions. EVs including exosomes encapsulate major matrix components and are emerging as promising diagnostic tumor biomarkers and therapeutic tools in oncology (Fig. 4B). This article focused on the influence of ECM biomechanical properties in mediating cell–cell communication, EVs release, and matrix‐based cell–cell communication during cancer development and progression. Future directions may involve the advanced biophysical characterization of the ECM to unravel its intricate properties. Targeted therapies that modulate ECM components or their interactions with cells hold promise for treatment approaches for diseases, such as cancer and fibrosis. Moreover, biomimetic ECM‐based bioscaffolds, accurately mimicking native tissue microenvironments, offer a controlled platform for studying cell behavior and facilitating tissue regeneration. Additionally, EVs/exosome‐based therapeutics hold potential for modulating ECM remodeling and cell–cell communication, particularly in cancer and fibrosis. Engineered EVs can be loaded with ECM‐modifying molecules, such as siRNAs, miRNA precursors/inhibitors, or signaling modulators to disrupt pro‐tumorigenic matrix components and pathways driving cell proliferation, EMT, invasion, and angiogenesis. Additionally, we propose that via targeted modulation (i.e., RTK inhibitors, CRISPR‐based gene editing) of intracellular signaling pathways, such as those involving TGF‐β, YAP/TAZ, or integrins, it may be possible to control the quantity and cargo of the cancer‐derived EVs/exosomes. This dual approach, based on reprogramming both EV/exosome content and secretion mechanisms, offers a dynamic strategy to remodel the TME. In fact, precision medicine approaches tailored to individual ECM profiles and disease characteristics may enhance treatment efficacy by utilizing advanced strategies to modulate the TME, targeting specific signaling pathways or incorporating immunomodulatory approaches. To this end, integrating multi‐omics approaches will be essential to comprehensively understand ECM‐mediated processes in health and disease by analyzing molecular interactions in a feedback‐responsive manner. Ultimately, translating fundamental discoveries into clinical applications throughout robust preclinical research is essential for the successful implementation of ECM‐targeted therapies toward personalized treatment approaches to attenuate cancer progression.

## Conflict of interest

The authors declare no conflict of interest.

## Author contributions

Z.P., S.M., N.E.K., C.K., and N.S.M. contributed to the interpretation and review of reported data, and writing – original draft. Z.P., S.M., and N.E.K. contributed to the writing – review and editing, and conceptual design and preparation of figures. M.F. contributed to the formal SEM analysis, formal analysis, acquisition, and data interpretation. N.K.K. contributed to the conceptual design, interpretation, and writing – review and editing. All authors have read and approved the final version for publication.
